# Hydrologic model predictability improves with spatially explicit calibration using remotely sensed evapotranspiration and biophysical parameters

**DOI:** 10.1016/j.jhydrol.2018.10.024

**Published:** 2018-12-01

**Authors:** Adnan Rajib, Grey R. Evenson, Heather E. Golden, Charles R. Lane

**Affiliations:** a Oak Ridge Institute for Science and Education, US Environmental Protection Agency, Office of Research and Development, Cincinnati, OH, USA; b Department of Food, Agricultural and Biological Engineering, Ohio State University, Columbus, OH, USA; c US Environmental Protection Agency, Office of Research and Development, National Exposure Research Laboratory, Cincinnati, OH, USA

**Keywords:** MODIS, Multi-objective, Calibration, Prairie Pothole Region, SWAT, Uncertainty

## Abstract

A hydrologic model, calibrated using only streamflow data, can produce acceptable streamflow simulation at the watershed outlet yet unrealistic representations of water balance across the landscape. Recent studies have demonstrated the potential of multi-objective calibration using remotely sensed evapotranspiration (ET) and gaged streamflow data to spatially improve the water balance. However, methodological clarity on how to “best” integrate ET data and model parameters in multi-objective model calibration to improve simulations is lacking. To address these limitations, we assessed how a spatially explicit, distributed calibration approach that uses (1) remotely sensed ET data from the Moderate Resolution Imaging Spectroradiometer (MODIS) and (2) frequently overlooked biophysical parameters can improve the overall predictability of two key components of the water balance: streamflow and ET at different locations throughout the watershed. We used the Soil and Water Assessment Tool (SWAT), previously modified to represent hydrologic transport and filling-spilling of landscape depressions, in a large watershed of the Prairie Pothole Region, United States. We employed a novel stepwise series of calibration experiments to isolate the effects (on streamflow and simulated ET) of integrating biophysical parameters and spatially explicit remotely sensed ET data into model calibration. Results suggest that the inclusion of biophysical parameters involving vegetation dynamics and energy utilization mechanisms tend to increase model accuracy. Furthermore, we found that using a lumped, versus a spatially explicit, approach for integrating ET into model calibration produces a sub-optimal model state with no potential improvement in model performance across large spatial scales. However, when we utilized the same MODIS ET datasets but calibrated each sub-basin in the spatially explicit approach, water yield prediction uncertainty decreased, including a distinct improvement in the temporal and spatial accuracy of simulated ET and streamflow. This further resulted in a more realistic simulation of vegetation growth when compared to MODIS Leaf-Area Index data. These findings afford critical insights into the efficient integration of remotely sensed “big data” into hydrologic modeling and associated watershed management decisions. Our approach can be generalized and potentially replicated using other hydrologic models and remotely sensed data resources – and in different geophysical settings of the globe.

## 1. Introduction

Calibration of a hydrologic model involves constraining the solution space, i.e., the range of parameter combinations, to identify the most optimal parameter set that ostensibly represents watershed physics. The traditional approach of calibration with gaged streamflow data may produce a pseudo-accurate model, showing “acceptable” skill for streamflow simulation at target locations while incorrectly representing internal watershed processes (Maneta et al., 2017; Rajib et al., 2016a, 2018a). Such a model, giving right answers for wrong reasons (a characteristic of equifinality; Beven, 2012; Beven and Freer, 2001; Favis-Mortlock, 2004), might lead to incorrect interpretation of a hypothesis or an unsuitable selection of land management alternatives for future scenarios. The potential availability of remotely sensed streamflow estimates at ungaged locations is alluring (NASA, 2016), but the information contained in streamflow time series may not sufficiently capture how vertical fluxes evolve at different spatial and temporal scales within the watershed (Birkel et al., 2014; Li et al., 2018). Against this backdrop, using spatially distributed remotely sensed estimates of water balance components (e.g., soil moisture and evapotranspiration) affords multi-scale, multi-objective calibration of hydrologic models (Bai et al., 2018; Fatichi et al., 2016; Li et al., 2016), which may help to remedy this equifinality, or pseudo-accuracy, issue.

While remotely sensed surface soil moisture is being increasingly used in model calibrations (e.g., Milzow et al., 2011; Li et al., 2018; Parajka et al., 2009; Sutanudjaja et al., 2014; Wanders et al., 2014), its influence on the simulation of root zone moisture, evapotranspiration and streamflow could be limited depending on model structure (Chen et al., 2011, Han et al., 2012). Several studies (e.g., Brocca et al., 2012; Rajib et al., 2016a; Silvestro et al., 2015) have recommended constraining models with remotely sensed estimate(s) that can represent the entire/majority portion of the root zone, biotic (e.g., vegetation growth) and abiotic (e.g., energy exchange) processes. This is where remotely sensed actual evapotranspiration (ET) emerge as an effective data resource (e.g., Herman et al., 2018; Immerzeel and Droogers; 2008; Kunnath-Poovakka et al., 2016; Rientjes et al., 2013; Vervoort et al., 2014; Winsemius et al., 2008).

Currently, two primary issues exist related to the use of remotely sensed ET in model calibration. First, reproducible methods for handling spatially distributed and widely available “big data”, such as remotely sensed ET, are not prominent in the literature. For example, inefficient methods for using remotely sensed ET data as part of a multi-objective calibration of hydrologic models across a large number of sub-basins (or any other spatial unit, e.g., grid-cells) may have resulted in the current perception that this approach improves ET but may have mixed or limited influence on streamflow simulations (e.g., López López et al., 2017; Tobin and Bennett, 2017). Second, most studies that have used remotely sensed ET data for model calibration focus only on calibrating surface, sub-surface and river routing parameters (e.g., Immerzeel and Droogers; 2008; Kunnath-Poovakka et al., 2016; Rientjes et al., 2013; Tobin and Bennett, 2017). This practice does not leverage the potential for biophysical parameters within a hydrologic model to improve the overall representation of its physical processes (Yang and Zhang, 2016; Zhu and Zhuang, 2015). It is yet to be explored whether biophysical parameters, such as those associated with vegetation dynamics, land-atmosphere interaction, and energy utilization, affect model calibration performance – with or without the use of remotely sensed ET data in calibration process. Our study fills these knowledge gaps using a modified version of the process-based semi-distributed hydrologic model Soil and Water Assessment Tool (SWAT) (Arnold et al., 2012; Gassman et al., 2007; Neitsch et al., 2011).

Through a series of calibration configurations, we investigate (i) the degree to which adding biophysical parameters not traditionally considered in a streamflow-only calibration effects hydrologic simulation, (ii) the difference in simulated hydrologic responses due to the inclusion of remotely sensed ET in a multi-objective calibration with gaged streamflow data, and (iii) the additional change in hydrologic responses if remotely sensed ET is utilized in a spatially distributed calibration approach. While this study methodologically benchmarks remote sensing-enabled SWAT calibration in a North American watershed, insights provided here should be applicable for other hydrologic models and geophysical settings as well.

## 2. Study area

**Fig. 1.**
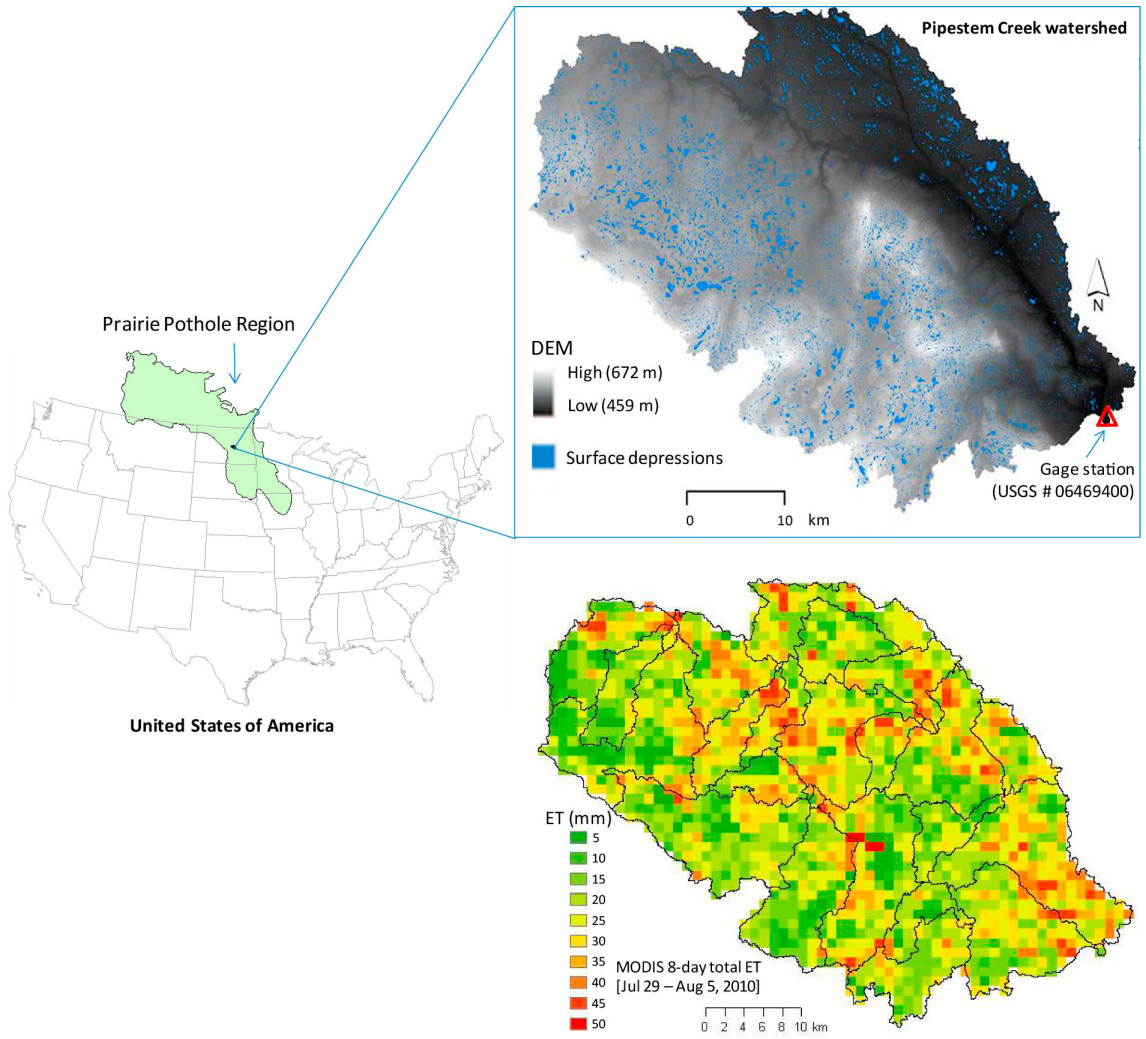
The Pipestem Creek watershed in Pingree, North Dakota, United States. The topographic map on top shows ~25,000 potential surface depressions; however, more than 50% of the watershed constitutes cropland and rangeland (Wu and Lane, 2016). The map at the bottom shows 29 sub-basins overlaid on 1 km MODIS grid; the example MODIS data represents total ET (mm) in an 8-day period (July 29 to August 5, 2010).

We conducted this study on the ~1670 km^2^ Pipestem Creek watershed in North Dakota, United States (Fig. 1). Located in the Prairie Pothole Region in the northern Great Plains, Pipestem Creek watershed landform consists of two ecoregions – the high-elevation Missouri Coteau in the west, and the Drift Plains in the eastern part of the watershed (EPA, 2011). The Coteau landscape in particular is pockmarked with thousands of wetland depressions, created by glacial retreat during the Wisconsinan period (Phillips et al., 2005; Winter and Rosenberry, 1995). These wetlands have been variously termed Prairie Potholes, including non-floodplain wetlands (NFWs) (Lane et al., 2018) amongst others (Tiner, 2003; Leibowitz, 2015; Mushet et al., 2015). Here, we will use the term surface depressions.

The surface depressions in the Prairie Pothole Region may be permanently inundated, ephemeral, or completely dry year-to-year (LaBaugh et al., 1998; Winter and Rosenberry, 1998). Primary water input in these surface depressions is the spring snowmelt and direct precipitation; as such water levels progressively drop through the summer months (Kantrud et al., 1989). This oscillatory dry-wet cycle makes ET a dominant driver of water, which responds to – and also affects – the “fluctuations and successions” of vegetation (Euliss et al., 2004; Gleason et al., 2008; van der Valk, 1981, 2005; van der Valk and Mushet, 2016; van der Kamp et al., 2016). With all these features, Pipestem Creek watershed is an ideal watershed for hydrologic modeling experiments.

## 3. Methodology

We constructed four calibration configurations of a modified SWAT model (Evenson et al., 2016) with increasing number of parameters and a progressively discretized way of optimizing them using remotely sensed ET data. The four configurations include:

- M1[Basic]: calibration using only streamflow data (Evenson et al., 2016);
- M2[+Biophysics]: another application of M1, incorporating biophysical parameters;
- M3[Lumped ET]: multi-objective calibration of M2 using both remotely sensed ET and streamflow data; parameters were optimized in a lumped approach commonly followed in many previous studies;
- M4[Spatial ET]: the same as M3, except the parameters were optimized in a spatially distributed approach.

Based on the outcome of these calibration experiments, we quantified the effects of biophysical parameters and remotely sensed ET data on simulated water balance components (specifically, ET and streamflow). To further reveal the nature of change in prediction uncertainty, temporal and spatial variability of streamflow, and vegetation growth, we have conducted additional assessments using two configurations. In the following, we first outline the model and the data used, then provide a detailed description of the model configurations.

### 3.1. A modified SWAT model

The major modeling components in SWAT include hydrology, vegetation dynamics, landscape and in-stream nutrient loading, stream temperature, and land management (Gassman et al., 2007). SWAT divides a watershed into multiple sub-basins, which are then further discretized into Hydrologic Response Units (HRUs) consisting of homogeneous land use, management, topographical, and soil characteristics (Neitsch et al., 2011). The modified SWAT model used in this study was originally developed by Evenson et al. (2016) with improved spatial representation and hydrologic functions. Evenson et al. (2016) conducted a series of novel modifications in the existing SWAT source code (Neitsch et al., 2011) to (i) enable a spatially explicit representation of surface depressions and hydrologic transport between surface depressions and uplands, (ii) improve seepage and sub-surface inflow simulation, and (iii) facilitate the simulation of fill-spill hydrologic relationships. There have been several applications of the SWAT model to specifically understand the hydrologic effects of surface depressions (e.g., Almendinger et al., 2011; Evenson et al., 2015, 2018; Liu et al., 2008; Rahman et al., 2016; Wang et al., 2008). SWAT has also been widely used for hydrological modeling in various geophysical settings worldwide, including major river basins in the United States (Rajib and Merwade, 2017), Europe (Abbaspour et al., 2015), Africa (Schuol et al., 2008) and Asia (Islam et al., 2017). Thus, findings from this study may be pertinent to worldwide SWAT model applications and at spatial scales much larger than the Pipestem Creek watershed.

### 3.2. Geospatial inputs and weather forcing

The 30-m National Elevation Dataset (USGS-NED, 2015), 30-m 2011 National Land Cover Database (NLCD) (Homer et al., 2015), and 1:250,000 State Soil Geographic Database (STATSGO) (NRCS, 2015; available within SWAT database) were the primary geospatial inputs in the modified SWAT model. To have a spatially explicit representation of surface depressions in the SWAT model, Evenson et al. (2016) first identified surface depressions using the National Wetland Inventory (NWI; http://www.fws.gov/wetlands/) and National Hydrography dataset (NHD; https://nhd.usgs.gov/) following Lane et al. (2012), and then estimated their storage capacities using a digital terrain constructed from 10-m NED (e.g., Lane and D’Amico (2010)). This approach provided unprecedented geospatial specificity to model a watershed as discretely as possible. Contrasting the 1217 HRUs that a SWAT model would produce for the Pipestem Creek watershed (based upon NED, NLCD, and STATSGO data), modified spatial representation by Evenson et al. (2016) had 62,721 HRUs within the same number of sub-basins (n = 29).

We used the Daymet daily total precipitation and average maximum-minimum temperature as the primary weather inputs (Thornton et al., 2014; gridded data available at 1-km spatial resolution), while creating ancillary weather inputs (i.e., solar radiation, wind speed and relative humidity) using the SWAT model’s built-in weather generator (WGN) (Neitsch et al., 2011). As we maintained identical weather inputs in all the four calibration configurations, possible propagation error resulted from Daymet and WGN data may have equivalent effect on the simulation results.

### 3.3. Mechanism for simulating ET and vegetation dynamics

We let SWAT estimate Potential Evapotranspiration (PET) using the Penman-Monteith (P-M) method such that ET simulations were compatible with MODIS (Moderate Resolution Imaging Spectroradiometer) algorithm (Section 3.4.2). Taking this PET as the maximum permissible limit, SWAT simulates an evaporative demand (ET_d_) that is spatially (across HRUs) and temporally (along daily time-steps) variable. SWAT’s P-M equation to estimate plant demand (the transpiration component of ET_d_) requires Leaf-Area Index (LAI). LAI, representing the spatio-temporal dynamics of vegetation growth, is simulated via a biome-specific semi-empirical leaf development curve with several user-defined biophysical parameters like radiation-use efficiency and maximum canopy height. Soil demand or the evaporation component of ET_d_ follows an exponential relationship with above-ground vegetation, also simulated by leveraging similar parameters (Rajib et al., 2018a). Eventually, SWAT uses the existing moisture content in soil profile to scale ETd into ET (Rajib and Merwade, 2016). This built-in architecture to simulate LAI and ET was retained in our modified SWAT model.

### 3.4. Reference data for model calibration and verification

#### 3.4.1. Streamflow

For calibration and verification of the modified SWAT model, we used streamflow data from the only available US Geological Survey (USGS) gaging station at the watershed outlet (Fig. 1), but varied how this data was included in the multi-objective calibration depending on the model configuration (see Section 3.5). This gage had 41% estimated values over a 5-year period (753 of 1826 data points between 2009 and 2013), especially prevalent during the winter months when icy conditions prevent gage reading. To avoid uncertainties implicit in statistical estimation of streamflow time-series (Chokmani et al., 2008; Hamilton, 2004), we only used the observed data in model calibration following Evenson et al. (2018 and 2016). However, given the large inter-annual hydrologic variability in the watershed (and the Prairie Pothole Region as a whole; van der Valk, 2005; van der Valk and Mushet, 2016; van der Kamp et al., 2016), it was deemed necessary to perform a long-term verification of the calibrated model leveraging the best available data. To facilitate this, we included both estimated and observed streamflow data for model verification purposes.

#### 3.4.2. Remotely sensed evapotranspiration and vegetation

The 8-day total, ~1 km gridded ET data from MODIS (MOD16A2 collection 5; Mu et al., 2013) was geo-referenced and spatially aggregated into each of the 29 sub-basins of the Pipestem Creek watershed using a semi-automatic web-based tool (Rajib et al., 2018b). While dynamically accounting for the heterogeneity in size, shape and locations of the sub-basins, the tool puts area-weighted average value of 8-day total ET (mm H_2_0) from encompassing and/or intersecting MODIS grid-cells onto each of the sub-basins. Despite potential inaccuracies due to spatial aggregation (Zhang et al., 2016), this areaweighted averaging is a reasonable scheme to create spatially explicit time-series of remotely sensed data for hydrologic modeling purposes (Ershadi et al., 2013; Rajib et al., 2018a).

To enable SWAT calibration at a daily time-scale, we temporally disaggregated the sub-basin level 8-day total MODIS ET data using the corresponding daily estimates from the North American Land Data Assimilation System (NLDAS- phase 2; NASA, 2017) (Eq. (1)):

(1) $$ET_{i(M)}=ET_{T(M)}\times \left[\frac{ET_{i(N)}}{ET_{T(N)}}\right]$$

here, *M* and *N* represent MODIS and NLDAS respectively; *T* refers to the total 8-day value; the index *i* denotes a particular day within that 8-day segment (*T*). We applied the same data-processor tool to create subbasin level NLDAS time-series from its original ~12 km gridded estimates. Both MODIS and NLDAS estimates are based on the Penman-Monteith (P-M) method, however, assumptions on aerodynamic/surface resistances as well as the sources of land use and meteorological input data in their respective algorithms are different. Differences between the absolute values of MODIS and NLDAS ET did not affect *ET*_*i*(*M*)_ as we used NLDAS estimate in Eq. (1) only as a temporal scaling factor, without altering the total amount of ET estimated by MODIS in each 8-day window (*ET*_*T*(*M*)_). Therefore, we labeled the MODIS-NLDAS hybrid estimate (*ET*_*i*(*M*)_) simply as MODIS ET.

Logic suggests that simulated ET will follow the spatio-temporal trend of reference ET that constrains a hydrologic model during the calibration process (MODIS data in our study). Therefore, to build multiple lines of evidence on the overall improvement of the model in representing watershed characteristics (and particularly the energy balance), we used LAI as an independent reference variable not included in the calibration process. Our LAI based evaluation is novel because it is the first to use vegetation dynamics for cross-verification when remotely sensed ET is applied in model calibration. Previous studies on model calibration using remotely sensed ET measured performance improvements based solely on simulated ET outputs (e.g., Herman et al., 2018; Immerzeel and Droogers; 2008; Rientjes et al., 2013; Tobin and Bennett, 2017) – no other variables were considered. To compare LAI simulations across model configurations, we obtained watershed-average LAI data from a 4-day total MODIS product (MCD15A3H version 6; Myneni et al., 2015). Creation of watershed-average LAI time-series from the original ~500 m gridded data again required employing the semi-automatic data-processor tool. However, no temporal re-scaling was necessary in this case as we computed 4-day total values from SWAT’s daily outputs to match temporal resolution of the original MODIS data.

With the advances in satellite sensor technologies and data assimilation techniques, various multi-source, robust ET products have emerged in recent years. Some of these relatively new ET estimates are available at a daily time-step, not requiring any temporal re-scaling/disaggregation (e.g., Zhang et al., 2016, 2010). Yet we preferred using MODIS ET and LAI data because these are open-source global datasets with long-term and continuous availability, reasonably good spatiotemporal resolution, and more importantly, well-documented quality assessments (e.g., Mu et al., 2011, 2013; Ruhoff et al., 2013; Yan et al., 2016; Yang et al., 2006; Velpuri et al., 2013). Because uncertainties are common in any remotely sensed product, none of these datasets should be considered as actual observations.

### 3.5. Calibration configurations

**Fig. 2.**
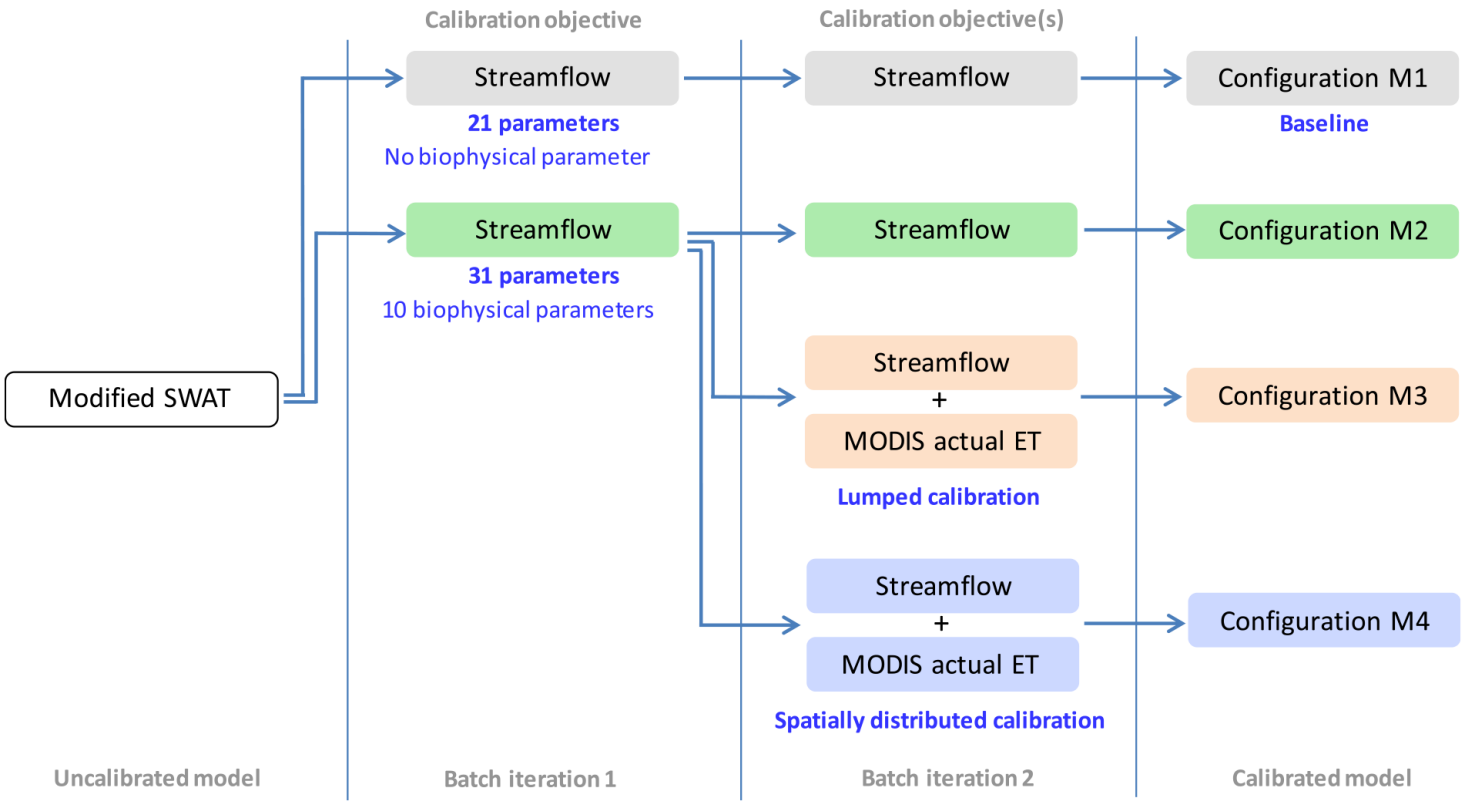
A schematic representation of calibration architecture and associated workflow involved in the four configurations. We reproduced the modified SWAT model developed by Evenson et al. (2016). Iteration 1 involved a simplistic sensitivity analysis to identify how inclusion of biophysical parameters might have affected the order of sensitivity (see Fig. 3). The most optimal model obtained through Iteration 1 became the starting point for Iteration 2. Calibration of snow parameters were discontinued after Iteration 1 in all cases (see Section 3.5.4 for details). Each batch of iterations (1 and 2) contains 250 simulations, however, the number is subjective.

Fig. 2 schematically outlines the modeling architecture involved in the four calibration configurations, namely M1[Basic], M2[+Biophysics], M3[Lumped ET], and M4[Spatial ET], all explained below in individual subsections. All calibrations were conducted at daily time-step using the Sequential Uncertainty Fitting algorithm (SUFI-version 2) in the SWAT-CUP platform (Abbaspour, 2015; Abbaspour et al., 2007). To be consistent with Evenson et al. (2018 and 2016), each configuration went through a 3-year initialization (2006–2008) followed by a 3-year calibration (2009–2011). The iterations were divided into two successive batches. SUFI-2 uses Latin Hypercube Sampling (LHS) to set potential parameter combinations based on the specified initial range for every calibration parameter. Using an inverse optimization approach, SUFI-2 then finds a narrower range of parameter values at the end of the preliminary batch to initiate the next batch for further iterations. We selected Kling-Gupta Efficiency (KGE; Gupta et al., 2009; Kling et al., 2012) as the objective function which was maximized (for streamflow and ET, depending on the model configuration) to find the most optimal parameter set. KGE decomposes Nash-Sutcliffe Efficiency (NSE) and Mean Squared Error (MSE) into a three-dimensional criteria space and finds out a Pareto front in terms of the shortest Euclidean distance (ED):

(2) $$KGE=1-ED=1-\sqrt{\{}{\left(r-1\right)}^{2}+{\left(\mu_{s}/\mu_{0}-1\right)}^{2}+{\left(\sigma_{s}/\sigma_{0}-1\right)}^{2}\}$$

where r represents the correlation, *μ*_s_*/μ*_0_ and *σ*_s_/*σ*_0_ respectively represent bias and variability ratio between the simulated and observed variable, μ and σ are the mean and standard deviation of the variable; the indices s and o denote simulation and observed data, respectively. KGE ranges from −∞ to 1, with a value closer to 1 indicates a relatively accurate model.

As noted above, our goal was to keep the total length of the simulation consistent with previously published research on Pipestem Creek watershed using the same modified model (e.g., Evenson et al., 2018, 2016). However, the need for long-term verification of model outputs and the limited availability of observed streamflow data (see Section 3.4.1) made it challenging to follow the conventional split-sample approach where model calibration and verification is conducted over two non-overlapping “independent” time-periods. We therefore evaluated (verified) our calibrated model configurations over a 5-year period (2009–2013). Despite this partial overlap with the calibration period (2009–2011), our approach resulted in an acceptable degree of independence among the calibration and verification datasets for three reasons: (1) we excluded estimated streamflow data from the model calibration but included them in the verification stage, (2) our model verification with streamflow and ET data included two more years (2012–2013) beyond the calibration period, and (3) we used LAI data as a measure of cross-verification. To facilitate further independent evaluation, and assess how a multi-objective use of ET and streamflow in SWAT calibration might change the model’s response during extreme flood events, we also excluded a specific portion of observed stream-flow record (August 2011) from the calibration period and used it for model verification.

#### 3.5.1. Baseline model (M1)

**Fig. 3.**
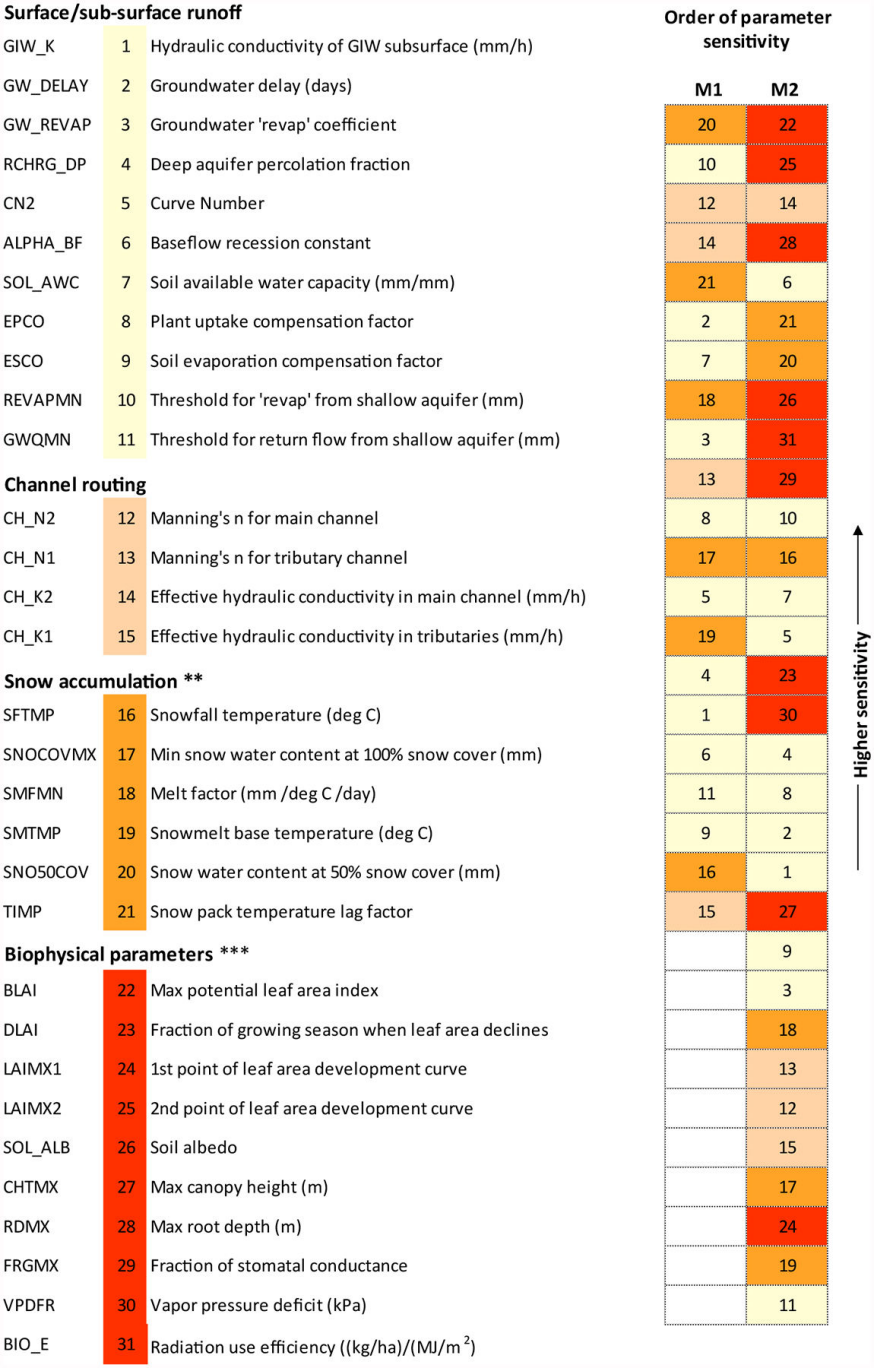
Calibration parameters and their order of sensitivity with and without the involvement of biophysical parameters. Respective initial ranges and the spatial scale of iteration are shown in Table A1 (Appendix A). See Neitsch et al. (2011) for parameter definitions, except GIW_K; GIW_K is a new SWAT parameter for surface depressions (Evenson et al., 2016). ** Calibration was discontinued after the first batch of iterations; *** these parameters were not calibrated in M1[Basic].

M1[Basic] involved calibration only with streamflow data at the watershed outlet, and included 21 parameters related to surface/subsurface runoff, channel routing, and snow accumulation processes in the calibration (Fig. 3 and Table A1 in Appendix A). All of these parameters, their respective initial range, and the M1 configuration as a whole, were identical with the calibration performed by Evenson et al. (2016), differing in this study only in the management of snow parameters. We excluded snow parameters after the first batch of iterations to be able to compare with M4 outputs (see below in Section 3.5.4). M1 was our baseline to measure the degree of model improvement in subsequent configurations.

#### 3.5.2. Effect of biophysical parameters (M2)

M2[+ Biophysics] was the same as M1[Basic], except with the addition of 10 parameters related to biophysical processes (see Fig. 3). There are more than 20 such parameters in SWAT geodatabase (Neitsch et al., 2011); however, they are often underutilized and assigned default values. The parameters we selected collectively represent a range of processes including vegetation growth, land-atmosphere feedback, and energy use mechanisms. Having more parameters would push the computational overhead of our hyper-resolution model (~63,000 HRUs) beyond a workable limit. Initial ranges for the 10 additional parameters were set following the suggestions from SWAT developers (Abbaspour, 2015).

Most of the contemporary SWAT applications, including those on watersheds with abundant surface depressions (e.g., Evenson et al., 2016; Rahman et al., 2016; Ikenberry et al., 2017), do not consider biophysical parameters in model calibration. The underlying notion is that biophysical parameters are least sensitive for hydrologic simulation compared to those representing runoff, routing and snow accu-mulation processes. However, due to the heavily conceptualized equations that SWAT employs to capture biophysical interactions, it is possible that including the relevant parameters within a simple streamflow-only calibration can make notable difference in the overall spatial hydrologic response of the model. We performed the built-in global sensitivity analysis scheme in SUFI-2 (Abbaspour, 2015; Rajib and Merwade, 2016) to substantiate this notion.

Optimizing 31 parameters through the first batch of iterations, the second batch in M2[+ Biophysics] excluded the snow parameters which made it comparable with M1[Basic] (see Fig. 2). Thus, differences in outputs between these two configurations identified the effect of biophysical parameters on model predictability.

#### 3.5.3. Use of remotely sensed ET data in model calibration (M3)

The most optimal model resulting from the first batch of iterations in M2[+ Biophysics] became the starting point for M3[Lumped ET] (Fig. 2). The difference between these two configurations was solely that M3[Lumped ET] included MODIS ET as an additional model constraint in the second (and final) batch of iterations. The optimization algorithm (SUFI-2) used 30 constraints together - 29 time-series of MODIS ET (one for each sub-basin) and a single time-series of streamflow data (watershed outlet). The 30 objective functions (essentially 30 KGEs), as shown in Eq. (3), were given equal weighting factor to form an aggregated value (KGE’):

(3) $$KGE^{\prime }=\sum_{i=1}^{n_{f}}w_{f_{i}}\left(KGE_{f_{i}}\right)+\sum_{j=1}^{n_{e}}w_{ej}\left(KGE_{ej}\right)$$

here, *n* and *w* are the number of objective variables (reference datasets) and the weight assigned to each of them, respectively; the values *f* and *e* stand for streamflow and ET, respectively. Also, *i* denotes the streamflow gaging stations and *j* denotes sub-basins with MODIS ET data. The parameter set producing the highest KGE′ was the most optimal one.

Unlike the runoff/routing parameters in SWAT, the majority of its biophysical parameters cannot be defined at HRU or sub-basin level. Instead, these parameters are associated with specific plant/biome type, and it is possible that the same biome type may exist in multiple HRUs or sub-basins. Because of this inconsistency, the M3 configuration restricts every parameter so that it can only be changed “in a unique way” irrespective of the HRU/sub-basin. More specifically, in successive iterations during the calibration, a particular parameter must have an identical amount/rate of change across the entire watershed, and that amount/rate is pre-selected from a Latin Hypercube Sampling of its initial range of values. This is essentially a lumped approach notwithstanding the inclusion of spatially distributed MODIS ET data in the calibration process (hereafter, referred as M3[Lumped ET]). Previous studies indiscriminately applied this lumped approach even though biophysical parameters were not included in their respective calibrations; consequently, it became common to lump all the available ET time-series into a single calibration target (Eq. (3)) (e.g., Immerzeel and Droogers; 2008; Rientjes et al., 2013; Tobin and Bennett, 2017).

Regardless of these limitations, M3[Lumped ET] configuration provided new insights. As noted, considering that the use of MODIS ET in M3[Lumped ET] was the only difference from M2[+ Biophysics], comparison of these two configurations can distinguish the effect of remotely sensed data from that of biophysical parameters which had not been addressed in any previous study.

#### 3.5.4. Value of spatially distributed calibration using remotely sensed ET (M4)

M3[Lumped ET] and M4[Spatial ET] were identical, except a “spatially distributed” calibration approach was implemented in the M4 configuration. The goal of M4[Spatial ET] was to leverage the maximum potential of spatially distributed MODIS ET data and allow highly discretized parameter optimization by calibrating 29 sub-basins individually. Each sub-basin had two constraints to find optimal parameters - a representative time-series of MODIS ET specific to the subbasin and a time-series of streamflow data at the watershed outlet.

Lack of spatial heterogeneity in snow accumulation processes is a limitation in the current SWAT modeling framework. All the snow parameters in SWAT can only be defined at watershed level (Neitsch et al., 2011). That is why iteration of snow parameters was technically impossible in a sub-basin level calibration approach such as M4[Spatial ET]. In order to have an even comparison with M4[Spatial ET], snow parameters were not adjusted after the first batch of iterations in any of the four configurations. This problem is very specific to SWAT; continued optimization of snow parameters throughout the calibration process, and that in an M4[Spatial ET]-type setup, might be doable in the case of other hydrologic models.

## 4. Results

### 4.1. Model predictability across the spectrum of calibration configurations

**Fig. 4.**
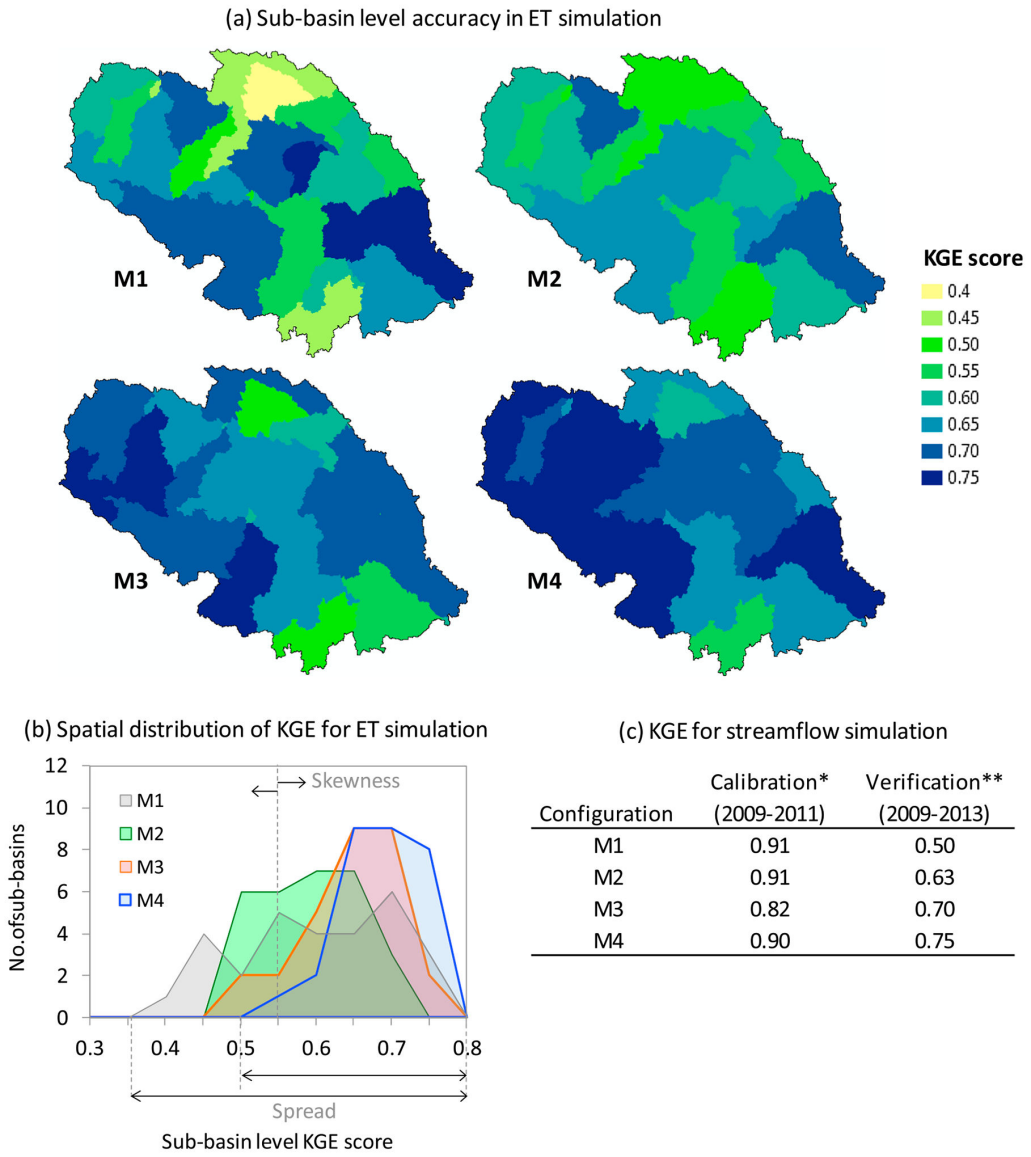
Goodness of model performance based on the most optimal parameter combination. KGE values shown here measure the agreement of the model with respect to MODIS ET at sub-basin level and USGS streamflow at the watershed outlet. All assessments are for daily simulation. While ET simulation performance shown here correspond only to the calibration period (2009–2011; Fig. 4a–b), streamflow simulation performance was reported both for calibration and verification (2009–2013; Fig. 4c): * using only the “observed” portion of the available USGS data; ** using both “observed” and “estimated” data (see Fig. A1 in appendix A for streamflow hydrographs).

Both biophysical parameters and remotely sensed ET data influenced model predictability. This is evidenced by the relative change of model performance among the four configurations, in terms of KGE for simulated daily ET at sub-basin level and streamflow at the watershed outlet (Fig. 4a–c). Considering the improved spatial representation, seepage/sub-surface inflow, and fill-spill hydrologic simulation of surface depressions (Evenson et al., 2016), the baseline configuration M1 should exhibit minimal inaccuracy in reproducing hydrologic processes. As expected, the high KGE score (0.90) in M1[Basic] for the most optimal streamflow simulation during the calibration period fit the traditional SWAT evaluation rubric of a “very good” hydrologic model (Moriasi et al., 2015). But pseudo-accuracy was evident based on the low watershed-average KGE (~0.60) for ET, demonstrating potential limitations of simulated vertical water fluxes in the M1[Basic] configuration. This may, in part, be an underlying reason that the streamflow simulation in M1[Basic] did not perform well in the verification phase (see Fig. 4c). The pseudo-accuracy was intriguing as it indicated the need for supplementing spatially explicit hydrologic transport between surface depressions and fill-spill mechanisms in a hydrologic model with measures that potentially account for other process uncertainties in the model. One way to address this issue was to focus on biophysical parameters. Inclusion of biophysical parameters in model calibration is appropriate because of the coupled relationships the vegetation and energy related processes have with the partitioning of water at different scales of a watershed.

The same model (M1[Basic]) showed a different, and slightly improved (see Fig. 4c), model performance simply when additional bio-physical parameters were included in a streamflow-only calibration (M2[+ Biophysics]). Compared to the variable spatial nature of ET simulation in the M1[Basic] configuration with sub-basins having very high (0.75) to very low (0.40) KGEs, M2[+ Biophysics] suggested an improved spatial accuracy via more consistent KGEs (0.50–0.70) across the watershed (Fig. 4b). This seemingly improved model state in M2[+ Biophysics] resonated in the streamflow verification showing relatively higher KGE than M1[Basic] (Fig. 4c). While these results confirmed the sensitivity of biophysical parameters to mediate hydro-logic processes in a watershed with abundant surface depressions and fill-spill phenomena (Fig. 3), one can expect that these parameters would affect hydrologic simulations in other geophysical settings too including agricultural and forested landscapes (e.g., Quinton et al., 2010; Yang and Zhang, 2016). However, it is expected that the cali-bration of biophysical parameters is more effective in the presence of constraint datasets “relevant” to their physical processes (e.g., ET and vegetation growth).

In the next configuration with MODIS ET as an additional calibration target (M3[Lumped ET]), we found considerable improvement in SWAT’s ET simulation (Fig. 4a and b). Hypothetically, improved ET implies improved physical representation of the model’s water balance components. As such, other hydrologic processes including streamflow should reflect traces of improvement too. This was evident in M3[Lumped ET] through a distinctively increased streamflow KGE during the model verification, although streamflow KGE decreased in the calibration period compared to M1[Basic] and M2[+ Biophysics] (Fig. 4c).

The goal of the M4[Spatial ET] configuration was to evaluate whether using the same calibration targets of M3[Lumped ET] in a spatially distributed approach can further improve model performance. Results from M4[Spatial ET] were the most telling in terms of KGE scores for both ET and streamflow simulations. The watershed-average KGE for ET was 0.70 in M4[Spatial ET], particularly contrasting to 0.60 in M1[Basic] and the highest among the four configurations. As we moved through our calibration experiments from one level to the next (M1 to M4), we identified increasing gain in the overall spatial accuracy of ET simulations (Fig. 4a and b). We interpreted this drift in spatial accuracy (“pulling of models”: Efstratiadis and Koutsoyiannis, 2010) by looking onto the spread and skewness of sub-basin level KGE distributions. For instance, the best spatial accuracy in M4[Spatial ET] (Fig. 4a) was reflected through the smallest spread (range of KGEs) and the maximum skewness (toward high KGEs) (Fig. 4b). This evolution of model performance quantitatively showed how the state of a model can transitionally improve in predictability and shrink in equifinality with a spatially explicit ET calibration.

In line with its spatial accuracy in ET simulation, M4[Spatial ET] showed remarkably improved streamflow simulation skill during the model verification (KGE = 0.75; Fig. 4c). While these results collecstively made M4[Spatial ET] the superior configuration, another key aspect made marked difference in the efficacy of M4’s spatially distributed calibration approach. Unlike M3[Lumped ET], M4[Spatial ET] did not show a drastic drop in streamflow calibration performance. It rather retained nearly the same calibration performance as M1[Basic] and M2[+ Biophysics], despite a multi-objective optimization with the same total volume of ET data used to calibrate M3[Lumped ET]. In this way, M4[Spatial ET] fully complied with the theoretical cause-and-effect expectancy that an improved representation of water and energy balance should result in improved streamflow simulation as well.

### 4.2. Additional insights on hydrologic representation

To further explore the overall improvements achievable through the M4[Spatial ET] configuration, below we present a holistic evaluation of its outputs focusing on three different perspectives: (i) uncertainty in predicted water balance, (ii) temporal and spatial variability of streamflow, and (iii) vegetation growth. Here, M4[Spatial ET] is contrasted only against M1[Basic] given these are the two extreme configurations in terms of model accuracy.

#### 4.2.1. Uncertainty in predicted water balance

**Fig. 5.**
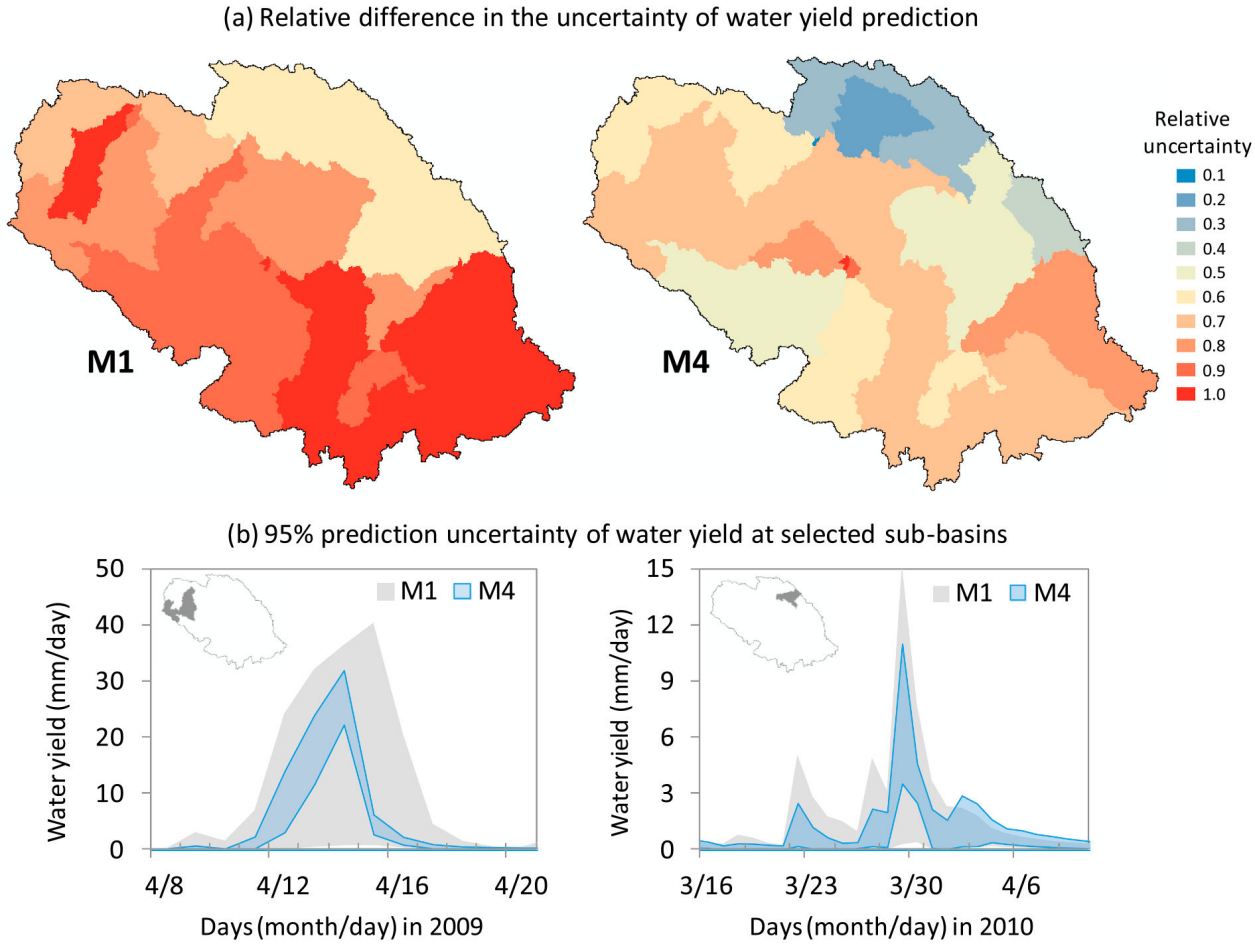
Sub-basin level assessment of water yield prediction uncertainty. The dimensionless relative uncertainty values mapped in (a) indicate average band width of 95% prediction uncertainty over a 3-year calibration period, normalized across space and irrespective of the configuration. Because of this normalization, these band width values measure the “relative” difference in prediction uncertainty between M1 and M4 (1 meaning the highest uncertainty). The band hydrographs in (b) compare 95% prediction uncertainty of water yield (mm/day) during two different peak flow events and two different physiographic regions within the watershed (high wetland density (left) and low wetland density (right)). We chose two different events to evaluate whether the reduced uncertainty in M4 was consistent regardless of the antecedent wetness condition and the peak flow magnitude.

M4[Spatial ET] showed substantially reduced uncertainty in simulated water yields compared to M1 (Fig. 5). We chose water yield as a holistic index of water balance because it is the amount of water contributed to stream after the model accounts for fill-spill hydrology and all hydrological losses (e.g., to evapotranspiration and soil infiltration; Neitsch et al., 2011). The relative uncertainty mapped in Fig. 5a reflects the average 95% prediction uncertainty (95ppu) band width in calibrated water yield outputs at the sub-basin level, normalized with respect to the minimum and maximum band widths across all the subbasins (irrespective of a particular configuration). Here, our simulations showed consistently smaller prediction uncertainty in M4[Spatial ET] irrespective of the location and timing of high flow events within the watershed (Fig. 5b).

#### 4.2.2. Temporal and spatial variability of streamflow

**Fig. 6.**
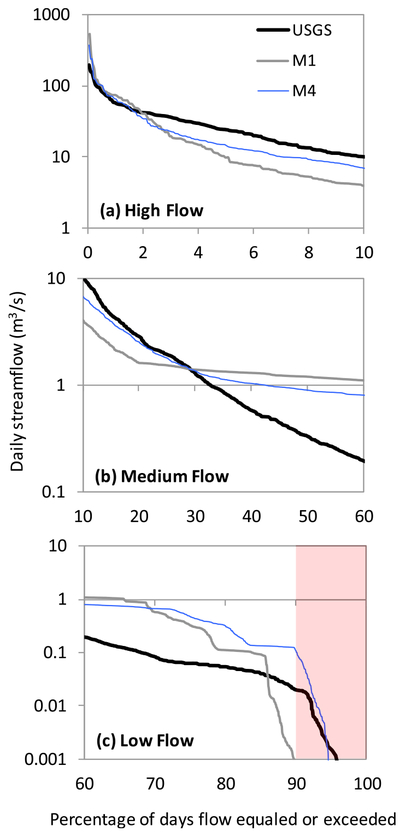
Model verification with USGS observed/estimated data for different exceedance probability of simulated streamflow at the watershed outlet during 2009–2013. Five different flow conditions (high, moist, mid-range, dry, and low) as defined in EPA (2007) were aggregated into three categories here: (a) high, (b) medium, and (c) low. Y-axis is in log_10_ scale. We compare the baseline model (M1) with our final model (M4) to convey the maximum contrast. Across all flow conditions, M4 simulations follow USGS data closer than M1, with exception of flows that are exceeded 20% of the time in the low flow condition (i.e., between 70 and 90% exceedance range; Fig. 6c). The highlighted portion in (c) indicates the prevalent watershed condition (~90% of the time during the 5-year simulation).

We compared the temporal variability of streamflow between M1[Basic] and M4[Spatial ET] in terms of their flow exceedance probability at the watershed outlet (Fig. 6). Irrespective of the flow regime (high flow, medium flow, and low flow), the most optimal streamflow output from M4[Spatial ET] generally showed better conformity with USGS data compared to M1[Basic]. Taking into account fill-spill processes during extreme events, improved high flow simulations in M4[Spatial ET] indicate enhanced functioning of the modified spatial representation and hydrologic transport proposed by Evenson et al. (2016) (Fig. 6). However, given the high inter-annual variability of ponded area in the Prairie Pothole Region (van der Valk and Mushet, 2016), it is not surprising that hydrologic models would struggle in reproducing average-condition water balance in the watershed. Accordingly, M1[Basic] and M4[Spatial ET] both resulted in large departures from USGS data during medium flow conditions, however, the difference (USGS *minus* SWAT) was consistently smaller in the case of M4[Spatial ET]. Furthermore, simulated streamflow in M4[Spatial ET] was in closer proximity with USGS data 90% of the time during the 2009–2013 period, indicated by the low flow conditions in Fig. 6. This was expected given the influence of ET on the diffusivity of soil moisture and mobility of groundwater when there is no surface runoff (Tobin and Bennett, 2017; Szilagyi et al., 2007). These results also afford temporal insight to the variation of streamflow KGE values shown in Fig. 4c.

**Fig. 7.**
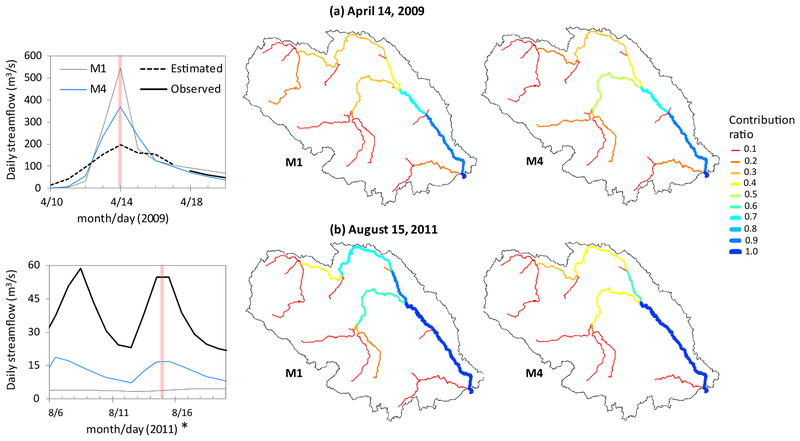
Relative contribution of tributaries and the main stem to the streamflow at the watershed outlet, comparing the M1 and M4 configurations. At a particular date, the so-called “Contribution Ratio” was calculated as the ratio of streamflow in a given stream segment to the streamflow at the outlet (e.g., 0.3 for a tributary indicates that 30% of the streamflow simulated at the watershed outlet is contributed by the particular tributary). To show consistently different landscape response in M4, we chose two different peak flow events: (a) a snow melt event on April 14, 2009 and (b) a rainfall event on August 15, 2011. In the respective events, peak flows in M1 and M4 configurations occurred at the same date (highlighted). In (b), * indicates a recorded flood event. USGS observed data from this particular date range was excluded from model calibration, but compared here with model simulations for cross-verification of the models’ goodness in responding to extreme events.

In addition to temporal dynamics, we found prominent differences between M1[Basic] and M4[Spatial ET] in the spatial variability of streamflow even when both configurations produced peak flows at the same time-stamp as the USGS data (Fig. 7). Apart from this identical temporal response particularly in the two specific flood dates shown here, we also noted the peak magnitudes which were distinctively more accurate in the case of M4[Spatial ET]. The combined impression from this duo (i.e., timing and magnitude) suggest that the spatial variability captured in M1[Basic] may have unrealistic representation of landscape response.

#### 4.2.3. Model accuracy in simulating vegetation growth

The traditional way of gaging the level of physical realism in a hydrologic model is to assess improvements in water balance components. Therefore, models are often not assessed for accuracy in energy balance components, although the energy balance can considerably influence water balance (Hurkmans et al., 2008; Rajib et al., 2018a; Troch et al., 2009). We considered watershed-average vegetation growth (LAI) as an indicator that implicitly represents the collective effects of improved water and energy balances in the model (Long et al., 2014).

**Fig. 8.**
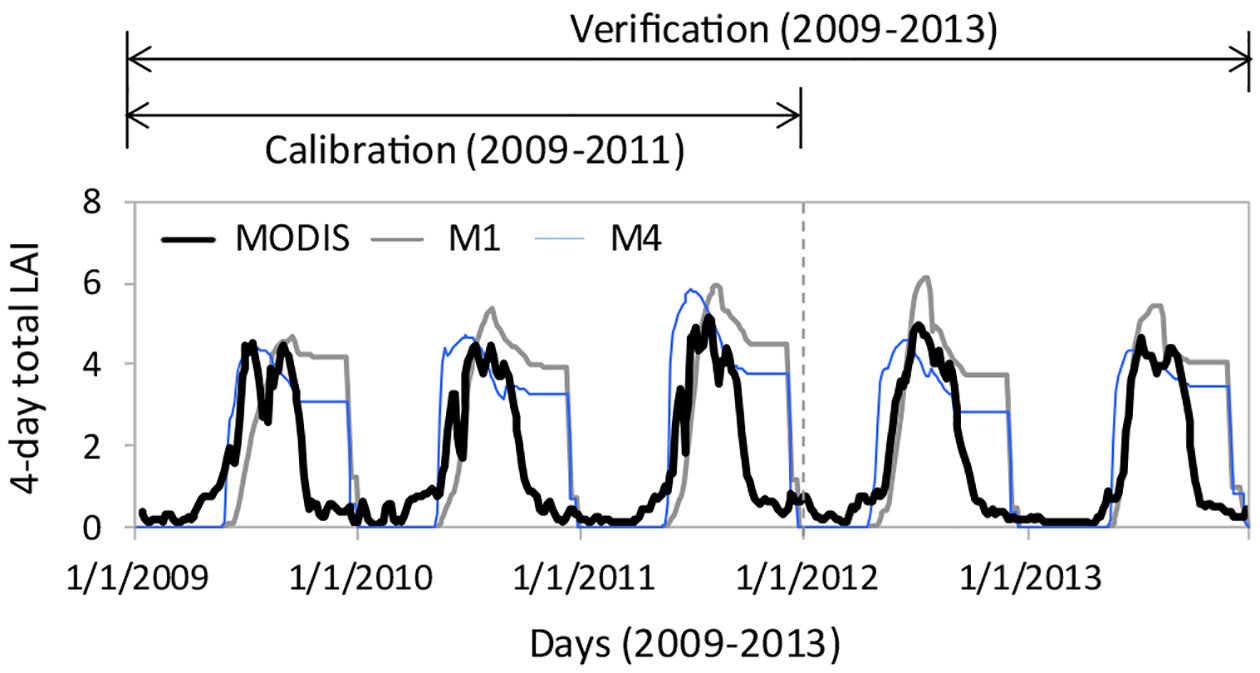
Comparison of simulated watershed-average LAI with corresponding MODIS 4-day total estimates. This is a cross-verification of the model because MODIS LAI was not part of our calibrations. LAI in M4 simulation follows MODIS data closer than M1.

M1[Basic] produced unrealistically high LAI values compared to M4[Spatial ET] and MODIS data (Fig. 8). The lack of biophysical parameters in the M1[Basic] calibration, and therefore the use of default values that, for the most part, stayed spatially constant across the watershed, undermined the ability of the model to simulate vegetation dynamics. The inaccurate LAI in M1[Basic] indicated another persistent limitation of the model suggesting that a modified water retentiondischarge (fill-spill) mechanism (Evenson et al., 2016) may only have limited influence on other hydrologic processes in a watershed. Although SWAT’s default LAI development formulation was not modified in any of our model configurations (based on SWAT’s internal structure; see Section 3.3), the parameter values defining the shape and inflection points of the LAI curve could be varied spatially in the M4[Spatial ET] calibration, and that using spatially explicit ET data. Therefore, the M4 LAI values compare better with the MODIS LAI. This improved representation of vegetation dynamics in M4[Spatial ET] compared to M1[Basic] was a reasonable indicator that the model was efficiently reproducing both water and energy partitioning, which may affect future biogeochemical applications of the model including carbon storage and nutrient cycling.

## 5. Discussion

### 5.1. Managing potential limitations involving remotely sensed data for calibration

Using remotely sensed data to calibrate a hydrologic model will inevitably produce different outputs compared to the same model that uses gaged streamflow data as the calibration objective. It is questionable, however, whether the differences in the remote sensing-integrated model emerge for right reasons. To enable physically meaningful application of remotely sensed ET in model calibration, and therefore get the right changes in model predictability for the right reasons, we took measures to overcome the following challenges.

#### 5.1.1. Is multi-objective calibration with ET and streamflow more effective, compared to using ET alone?

Simultaneous use of streamflow and ET in hydrologic model calibration is recommended as a “best practice” (e.g., Kunnath-Poovakka et al., 2016; Rientjes et al., 2013; Vervoort et al., 2014). Studies using remotely sensed soil moisture preferred the same (Li et al., 2018; Rajib et al., 2016a; Wanders et al., 2014). A model calibrated against only ET or soil moisture would produce too little or too much of a particular vertical water flux (to the atmosphere or through soil horizons) unless the horizontal water-routing in the model are also simultaneously adjusted. The outcome would be a highly equifinal model having good accuracy for a specific vertical flux but adverse or no change in streamflow simulation accuracy (e.g., López López et al., 2017; Tobin and Bennett, 2017; Wanders et al., 2014). For similar reasons, decoupling the horizontal water-routing component is a potential reason why some current-generation hybrid land surface-river routing models exhibit acknowledged timing problems in vertical fluxes as well as in streamflow simulations (e.g., Lin et al., 2018). Therefore, it is rational to accept that watershed hydrologic responses can be best captured when both the vertical and the horizontal flux components in the model are constrained with relevant calibration targets, such as ET and streamflow.

The amount of uncertainty in remotely sensed ET is purportedly higher than that in gaged streamflow data (Vervoort et al., 2014). In our initial exploration (not shown), we found that MODIS ET, if used from the very beginning of the calibration process, inserts propagating error so that the accuracy of simulated streamflow (with respect to gage station data) cannot be improved beyond a certain level. We also found that this model state is irreversible and may not be reinstated without changing the sequence of introducing MODIS ET data into the calibration process. Therefore, we hypothesized that calibrating the model first with gaged streamflow, a relatively well-verified in-situ dataset, produces a reasonable water balance that could serve as a baseline for further adjustment of hydrologic fluxes through a multi-objective approach involving both streamflow *and* ET. This two-step calibration is also favorable for applying other remotely sensed datasets including soil moisture for which “systematic bias” is a deterring factor (Draper et al., 2009; Reichle and Koster, 2004). Simulated soil moisture output from a model that is pre-calibrated against streamflow data can be used to reduce systematic bias in the remotely sensed soil moisture before it is integrated with the model through a data assimilation and/or a recalibration process (e.g., López López et al., 2017; Rajib et al., 2016a; Wanders et al., 2014).

#### 5.1.2. Is it justified to calibrate ET-related parameters separately?

Related to the two-step calibration explained above, it may not be physically coherent to calibrate biophysical or ET-related parameters separately. Herman et al. (2018) applied remotely sensed ET data to adjust only four additional parameters in a SWAT model, excluding all the other surface, subsurface, and routing parameters that were initially calibrated using streamflow data in a preceding step. As we performed global sensitivity analysis, several parameters with apparent high sensitivity in M1[Basic] (e.g., main channel Manning’s n) were ultimately found to be less sensitive in the presence of biophysical parameters (see Fig. 3). This provides supportive evidence that parameters in processbased hydrologic models (e.g., SWAT) or coupled atmospheric-land surface models (e.g., WRF-Hydro, Gochis et al., 2015) represent an integrated natural system. As such, they have an unknown degree of mutual relationships that may not be explicitly evident from their mathematical equations (Sivakumar and Singh, 2012). Therefore, not using a common set of parameters throughout the calibration process convolutes the effect of a new objective function (remotely sensed data) with that of the additional parameters initially not considered in preceding iterations. Such a calibration protocol may not be ideal, because it cannot identify whether the improvement in model simulation is solely due to the use of remotely sensed data.

### 5.2. Benchmarking a calibration strategy using spatially distributed remotely sensed data

The substantial disparity of simulation accuracy in M4[Spatial ET], compared to M1[Basic], M2[+ Biophysics] or M3[Lumped ET], ratio-nalizes the “spatially distributed use” of remotely sensed ET data in hydrologic model calibration. While this corroborates suggestions made in previous literature (e.g., Immerzeel and Droogers, 2008; Rientjes et al., 2013; Tobin and Bennett, 2017), assessments in these studies were mostly based on an M3[Lumped ET]-type configuration. Our study is the first to identify that M3[Lumped ET] produces an ostensibly good but actually sub-optimal model state. It is therefore worthwhile to review what factors undermine M3[Lumped ET] so that one can benchmark M4[Spatial ET] as a better approach for model calibrations, especially on large spatial scales.

The sub-optimality of M3[Lumped ET] was apparent mainly through the distinctive decrease in its streamflow calibration performance, notwithstanding the improvement it offers in ET simulation (Section 4.1). Importantly, Herman et al. (2018) showed similar tendency with a number of optimization algorithms including Non-dominated Sorted Genetic Algorithm II and Monte Carlo Simulations. It could also be a problem specific to the source of our data (MODIS ET; Mu et al., 2011, 2013), considering the uncertainty commonly seen in all remotely sensed estimates. However, previous studies reported similar results calibrating respective hydrologic models with ET data from the Surface Energy Balance System (SEBS) (Rientjes et al., 2013), Atmosphere-Land Exchange Inverse (ALEXI) model (Herman et al., 2018), and Global Land Evaporation-Amsterdam Model (GLEAM) (López López et al., 2017; Tobin and Bennett, 2017). This was evident when models were calibrated with soil moisture data as well (Li et al., 2018; Rajib et al., 2016a; Wanders et al., 2014). Given the above, the sub-optimality problem does not seem fundamental to the data, model or optimization algorithm.

We suggest that the sub-optimality in M3[Lumped ET] was, in part, an implementation problem in this configuration’s set-up that requires the optimization algorithm to handle too many objectives simultaneously. In the context of this study, the SUFI-2 parameter identification program resulted in an “averaging effect” while searching for a parameter combination that was optimal for all the 30 objectives (or constraints; Section 3.5.3; Eq. (3)). What had not been addressed in the existing literature is the inefficient use of emerging earth data resources and/or inappropriate handling of model parameters (e.g., Herman et al., 2018; López López et al., 2017; Tobin and Bennett, 2017; see Section 5.1), which are also viable factors affecting model performance in general.

The potential explanations for the limited performance or sub-optimality in M3[Lumped ET] have contributed to an evolving misperception that calibration with remotely sensed ET or soil moisture data may not improve streamflow simulations. As a result, there is a growing number of remote sensing studies that discourage the use of “lowered streamflow calibration performance” as a generic goodnessmetric to label a model’s overall streamflow prediction skills (Kunnath-Poovakka et al., 2016; Rajib et al., 2016a; Rientjes et al., 2013; Wanders et al., 2014). Li et al. (2018), in a multi-objective calibration using soil moisture and streamflow, reported substantially increased streamflow accuracy when their most optimal model was verified at locations that were not included in the calibration process, despite an apparently poor performance in target calibration locations. This was analogous to our M3[Lumped ET] results, which had a higher KGE value than M1[Basic] and M2[+ Biophysics] in the streamflow verification process (Fig. 4c), indicating model improvement in the right direction. Nonetheless, continued applications of M3[Lumped ET], given what our results suggest, could produce acute problems in large scale hydrologic modeling (e.g., Emerton et al., 2016; Lin et al., 2018). With more objectives to target and sub-basins to calibrate (i.e., a very large number of streamflow and ET time-series), M3[Lumped ET] might result in diminishing returns with no net improvement in water balance.

The M4[Spatial ET] configuration moves beyond the “averaging effect” issue, even though it uses the same total volume of MODIS datasets as M3[Lumped ET]. We therefore considered M4[Spatial ET] an improved approach compared to M3[Lumped ET]. However, it is computationally expensive to operate an M4[Spatial ET]-type setup and harness the maximum capacity of spatially distributed data by calibrating each of the sub-basins individually. At this point, we could argue that one can still adopt M3[Lumped ET] and make a tradeoff by accepting a calibrated model that is generally representative of internal watershed processes but relatively poor in streamflow simulation. Yet this argument lacks scientific integrity because it gives an impression that models with persistent problems are acceptable. The tradeoff argument also weakens the value of remote sensing integrated hydrologic modeling amidst the new trend of large scale streamflow/flood forecasting initiatives world-wide (e.g., Li et al., 2016; Lin et al., 2018). With the recent advancements in open-access, cyber-enabled, high performance calibration platforms such as SWATShare (Rajib et al., 2016b) and their potential interoperability with observational data resources (Horsburgh et al., 2016; Morsy et al., 2017; Tarboton et al., 2014), developing a computationally efficient reproducible workflow for the M4[Spatial ET]-type calibration configuration is no more impossible.

## 6. Summary and conclusion

It is not surprising that a hydrologic model may misrepresent the true state of water and energy balances given the complexity involved in simulating watershed hydrology correctly. One approach that has been increasingly used to improve simulation accuracy, particularly in watersheds with abundant surface depressions, is the modification of existing model frameworks to improve spatially explicit hydrologic transport between and among surface depressions and other water bodies, as well as retention-discharge (fill-spill) mechanisms. In this study, we hypothesized that the accuracy of such a modified model can be further improved by including biophysical parameters and remotely sensed ET data in the model calibration process. Specifically, we constructed four calibration configurations of a modified SWAT model, namely M1[Basic], M2[+ Biophysics], M3[Lumped ET] and M4[Spatial ET], with increasing number of parameters and a progressively discretized way of optimizing them using MODIS ET data. Based on the outcome of this experiment, the following conclusions were drawn.

1. Despite modifying the hydrologic transport and water retentiondischarge (fill-spill) mechanism, calibration of a SWAT model involving the traditionally used runoff-routing parameters and using only streamflow data (M1[Basic]) was less accurate in its simulated water balance components (namely ET and streamflow) than the other configurations with improved calibration methods (M2-M4).
2. The same model showed slightly improved ET and streamflow simulations when biophysical parameters were included in the calibration process (M2[+ Biophysics]). While this finding confirms the sensitivity of biophysical parameters, we suggest that their calibration would be effective (and meaningful) if the model is constrained with datasets that are more relevant to vegetation dynamics, land-atmosphere interaction, and energy utilization processes (e.g., ET and/or LAI).
3. Regardless of the lumped nature of calibration, we found that the M3[Lumped ET] (i.e., the M2[+ Biophysics model calibrated with sub-basin MODIS ET data and gaged streamflow aggregated as a single optimization objective) noticeably increased the accuracy of SWAT’s ET simulation compared to M1[Basic] and M2[+ Biophysics]. This configuration also resulted in improved streamflow simulations during model verification, indicating a transition of the model state towards increased predictability and reduced equifinality. Despite the seemingly improved water and energy balance, streamflow calibration performance in M3[Lumped ET] was distinctively lower than M1[Basic] and M2[+ Biophysics] configurations. We argued that this sub-optimality problem is not specific to a model, remotely sensed dataset or optimization algorithm. Instead, it is an implementation problem due to the aggregation of too many objectives, which imparts an “averaging effect” on the optimized solution. This may end up producing no net improvement of the model in large spatial scales.
4. Our results confirmed the superiority of M4[Spatial ET] (i.e., the model configuration with a spatially distributed approach such that each sub-basin was calibrated individually) over M3[Lumped ET] for overall model accuracy, although both configurations used the same total volume of remotely sensed data. Further, a one-to-one comparison between the two extreme configurations (i.e., M4[Spatial ET] versus M1[Basic]) indicated that an M4-type setup may exhibit reduced uncertainty in the simulation of landscape’s water yield, with distinctly different temporal and spatial variability of streamflow and more accurate representations of vegetation growth with respect to MODIS LAI data. We therefore conclude that modifying certain process descriptions of a hydrologic model (e.g., hydrologic transport between surface depressions, fill-spill mechanism) (M1[Basic]) may not sufficiently improve overall partitioning of water and energy in a watershed unless the model is calibrated with remotely sensed ET data, and that including biophysical parameters (M4[Spatial ET]).

The substantial disparity between the two extreme configurations (i.e., M4[Spatial ET] versus M1[Basic]) bears immense practical implications. The choice of model configurations can impact how scientists and managers quantify and interpret the hydrological and biogeochemical influence of surface depressions under different climate, land use, and potential depression loss/restoration scenarios. Using M1[Basic] for modeling landscape hydrological and nutrient hotspots/hot moments may lead to inefficient watershed management decisions. Therefore, we suggest that the spatially distributed calibration approach using remotely sensed ET data and including important biophysical parameters (M4[Spatial ET]) will lead to a more accurately calibrated model that does improve the representation of a watershed’s water and energy balance. While our methodological insights benchmarked efficient utilization of remotely sensed big data, the findings further provided scientific evidence on the evolution of a hydrologic model in the continuum of equifinality.

## Figures and Tables

**Fig. A1.**
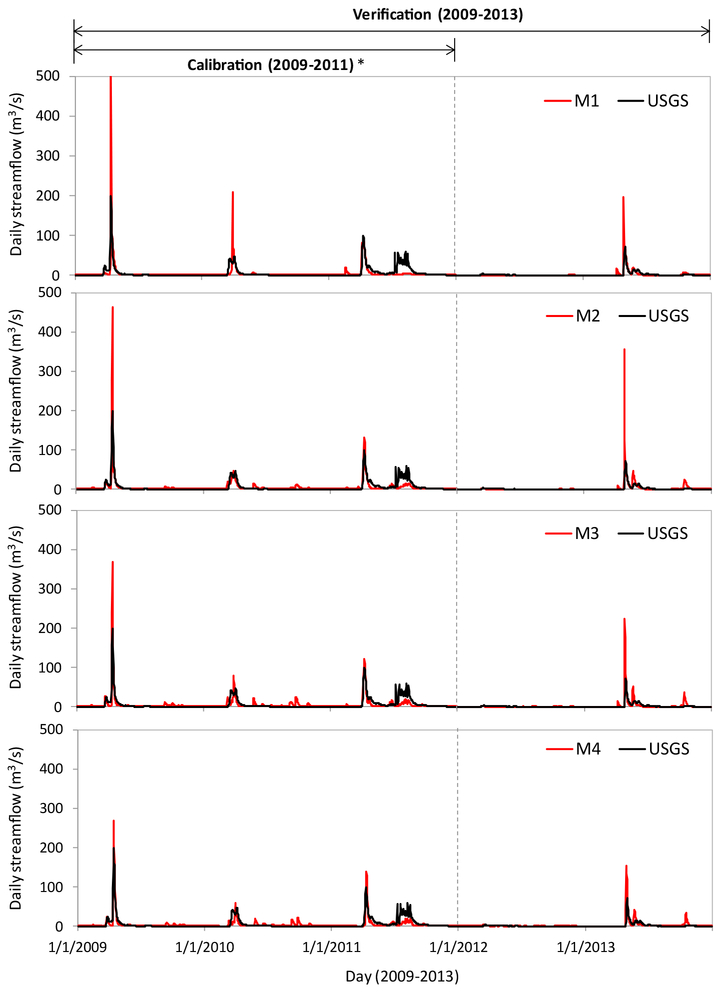
Comparison of simulated streamflow with USGS data at the watershed outlet during 2009–2013: * the “estimated” portion of the available USGS data were not included in calibration (see Sections 3.4.1 and 3.5 for details). Simulated hydrograph in a calibration configuration (M1-M4) corresponds to respective most optimal parameter combination. Goodness measures of model performance are shown in Fig. 4c.

## Appendix A

**Table A1:** Calibration parameters, their respective initial ranges and the spatial scale of iteration. All parameters, except CN2 and SOL_AWC, were varied such that the original parameter value gets replaced by another value within the assigned range. CN2 and SOL_AWC were adjusted with a multiplying factor relative to the original value. Calibration of snow parameters was discontinued after the first batch of iterations. Biophysical parameters were not calibrated in the M1[Basic] configuration. See Section 3.5 and Fig. 2 for details.

No.	Parameter	Name	Spatial scale	Initial range
*Surface/sub-surface runoff*				
1	GIW_K	Hydraulic conductivity of GIW subsurface (mm/h)	HRU	0–3.6
2	GW_DELAY	Groundwater delay (days)	HRU	0–500
3	GW_REVAP	Groundwater ‘revap’ coefficient	HRU	0.02–0.2
4	RCHRG_DP	Deep aquifer percolation fraction	HRU	0–1
5	CN2	Curve Number	HRU	−0.15–0.15
6	ALPHA_BF	Baseflow recession constant	HRU	0–1
7	SOLAWC	Soil available water capacity (mm/mm)	HRU	−0.2–0.2
8	EPCO	Plant uptake compensation factor	Basin	0–1
9	ESCO	Soil evaporation compensation factor	Basin	0–1
10	REVAPMN	Threshold for ‘revap’ from shallow aquifer (mm)	HRU	0–500
11	GWQMN	Threshold for return flow from shallow aquifer (mm)	HRU	0–5000
*Channel routing*				
12	CH_N2	Manning’s n for main channel	Sub-basin/Reach	0.01–0.3
13	CH_N1	Manning’s n for tributary channel	Sub-basin/Reach	0.01–0.3
14	CH_K2	Effective hydraulic conductivity in main channel (mm/h)	Sub-basin/Reach	0–150
15	CH_K1	Effective hydraulic conductivity in tributaries (mm/h)	Sub-basin/Reach	0–150
*Snow accumulation*				
16	SFTMP	Snowfall temperature (deg C)	Basin	−1.5–1
17	SNOCOVMX	Min snow water content at 100% snow cover (mm)	Basin	5–35
18	SMFMN	Melt factor (mm/deg C/day)	Basin	1.4–6.9
19	SMTMP	Snowmelt base temperature (deg C)	Basin	0–3
20	SNO50COV	Snow water content at 50% snow cover (mm)	Basin	0.05–0.35
21	TIMP	Snow pack temperature lag factor	Basin	0–1
*Biophysical parameters*				
22	BLAI	Maximum potential leaf area index	Biome-specific	0.5–10
23	DLAI	Fraction of growing season when leaf area declines	Biome-specific	0.15–1
24	LAIMX1	1st point of leaf area development curve	Biome-specific	0.001–1
25	LAIMX2	2nd point of leaf area development curve	Biome-specific	0.001–1
26	SOL_ALB	Soil albedo	HRU	0.001–0.25
27	CHTMX	Max canopy height (m)	Biome-specific	0.1–20
28	RDMX	Max root depth (m)	Biome-specific	0.001–3
29	FRGMX	Fraction of stomatal conductance	Biome-specific	0.001–1
30	VPDFR	Vapor pressure deficit (kPa)	Biome-specific	1.5–6
31	BIO_E	Radiation use efficiency ((kg/ha)/(MJ/m2)	Biome-specific	10–90
